# Substrate Stiffness Regulates Proinflammatory Mediator Production through TLR4 Activity in Macrophages

**DOI:** 10.1371/journal.pone.0145813

**Published:** 2015-12-28

**Authors:** Michelle L. Previtera, Amitabha Sengupta

**Affiliations:** 1 JFK Neuroscience Institute, JFK Medical Center, 65 James Street, Edison, New Jersey, 08820, United States of America; 2 Department of Neuroscience, Seton Hall University, 400 South Orange Avenue, South Orange, New Jersey, 07079, United States of America; Mayo Clinic College of Medicine, UNITED STATES

## Abstract

Clinical data show that disease adversely affects tissue elasticity or stiffness. While macrophage activity plays a critical role in driving disease pathology, there are limited data available on the effects of tissue stiffness on macrophage activity. In this study, the effects of substrate stiffness on inflammatory mediator production by macrophages were investigated. Bone marrow–derived macrophages were grown on polyacrylamide gels that mimicked the stiffness of a variety of soft biological tissues. Overall, macrophages grown on soft substrates produced less proinflammatory mediators than macrophages grown on stiff substrates when the endotoxin LPS was added to media. In addition, the pathways involved in stiffness–regulated proinflammation were investigated. The TLR4 signaling pathway was examined by evaluating TLR4, p–NF–κB p65, MyD88, and p–IκBα expression as well as p–NF–κB p65 translocation. Expression and translocation of the various signaling molecules were higher in macrophages grown on stiff substrates than on soft substrates. Furthermore, TLR4 knockout experiments showed that TLR4 activity enhanced proinflammation on stiff substrates. In conclusion, these results suggest that proinflammatory mediator production initiated by TLR4 is mechanically regulated in macrophages.

## Introduction

Biophysical changes in tissues correlate or and contribute to disease progression [1,2,3,4,5,6,7]. For example, clinical data and animal models have shown alterations in tissue stiffness are associated with multiple sclerosis [5], cancer [6,7], atherosclerosis [3], cardiovascular disease [3,4], and liver disease [1,2]. While deregulation of inflammation exacerbates disease pathogenesis, little is known about the influences of disease–related tissue stiffness changes on inflammation.

Macrophage activity plays a critical role in disease pathogenesis. Macrophages are white blood cells of the innate immune system that release proinflammatory and anti-inflammatory cytokines to orchestrate acute and chronic inflammatory events. Macrophages exist in a steady state in every healthy tissue including the liver, lungs, lymphoid organs, gastrointestinal tract, central nervous system, serous cavities, bone, and skin [8,9]. This means that steady–state macrophages are exposed to a variable range of tissue stiffnesses from extremely soft tissues, such as fat (17 Pa), to extremely stiff tissues, such as tendons (310 MPa) [10].

Several studies have addressed the effect of substrate stiffness on macrophage activity. Patel *et al*. grew U937 and RAW264.7 in the presence of LPS on 0.3–76.8 kPa polyacrylamide hydrogels (PA gels) [11]. Irwin *et al*. grew differentiated THP-1 cells attached to IPNs with moduli of 1.4–348 kPa [12]. Both reports showed that substrate stiffness was inversely proportional to TNF–α production. Interestingly, Blakney *et al*. reported that bone marrow–derived macrophages (BMMs) in the presence of LPS produced more TNF-α, IL–10, IL–6, and IL–1β when grown on 840 kPa poly(ethylene glycol)–RGD gels than when grown on 130 kPa or 240 gels [13]. Together, these reports suggest that the effect of substrate stiffness on macrophage activity is unclear. Studies need to investigate not only inflammatory signatures of macrophages grown on elastic substrates, but also investigate the inflammatory signaling pathways to address the effect of substrate stiffness on macrophage behavior.

Macrophages express toll–like receptor 4 (TLR4) in numerous diseases where tissue stiffness is altered including cancer, atherosclerosis, and cardiovascular disease [14]. Ligands, such as lipopolysaccharide (LPS), are known to activate the TLR4 signaling pathway to initiate the production of inflammatory mediators Investigations focus on identifying the ligands, such as LPS, that regulate TLR4 activity in diseased tissues [15,16,17,18]. However, limited studies have considered that tissue stiffness may regulate TLR4 activity.

In this report, we aim to elucidate the effect of substrate stiffness on macrophage proinflammatory mediator production as well as on TLR4 activity to determine whether substrate stiffness regulates the proinflammatory signaling cascades. To that end, we plated BMMs on PA gels, which mimicked a wide range of tissue stiffnesses, and stimulated BMMs with LPS. We evaluated proinflammatory mediator production in stimulated and unstimulated (US) BMMs. We found that substrate stiffness influenced proinflammatory mediator production. Next, TLR4 activity and the activity of downstream signaling molecules were evaluated to determine the effect of substrate stiffness on TLR4 signal transduction. Our data suggest that TLR4 signal transduction is regulated by substrate stiffness when macrophages are stimulated with LPS.

## Materials and Methods

### Antibodies

Rabbit polyclonal anti–TLR4, IκBα, p–IκBα, and anti–IFN–β antibodies were purchased from GeneTex (Irvine, CA) or Santa Cruz Biotechnology (Santa Cruz, CA). Rabbit polyclonal anti–GAPDH antibody was purchased from Sigma–Aldrich (St. Louis, MO). Rabbit anti–NF–κB, phosphorylated NF–κB p65 (p–NF–κB), TNF–α, IL–1β, and IL–6 antibodies were purchased from Cell Signaling Technology (Danvers, MA). Rabbit anti–iNOS was purchased from Abcam (Cambridge, MA). Goat anti–rabbit poly–HRP was purchased from Pierce (Rockford, IL). Alexa Fluor 594 anti–rabbit was purchased from Invitrogen (Carlsbad, CA).

### PA gel fabrication and functionalization

0.3, 1, 6, 27, 47, 120, and 230 kPa PA gels were made and functionalized as previously described [19]. Gels were functionalized by UV irradiation of Sulfo–SANPAH (0.315 mg/ml in water, ProteoChem, Loves Park, IL). Gels were incubated with poly–D–lysine (PDL, 0.1 mg/ml in water, Sigma–Aldrich), laminin (0.08 mg/ml in water Sigma–Aldrich), or collagen (0.2 mg/ml in 0.2 N acetic acid, Elastin Products, Owensville, MO) overnight at 4°C.

### BMM cultures

All animal protocols were approved by the Institutional Animal Care and Use Committee, a shared committee between Seton Hall University and the JFK Neuroscience Institute at JFK Medical Center. Mice were anesthetized with isoflurane and then euthanized by cervical dislocation. Monocytes were harvested and differentiated into macrophages, as previously described [20]. Briefly, bone marrow cells were harvested from the tibias and femurs of female, C57BL/6 and B6.B10ScN^-Tlr4lps-del^/JthJ (TLR4–deficient, Jackson Laboratory, Bar Harbor, ME) mice. Cells were plated onto non–tissue culture treated petri dishes and differentiated into macrophages by culturing in DMEM/F12 media supplemented with 10% fetal bovine serum, 1% penicillin/streptomycin, and 20–40 ng/ml of macrophage–colony stimulating factor (eBioscience, San Diego, CA). After 6–7 days, macrophages were trypsinized and all gels were plated at the same density, 50,000 cells/cm^2^. In most cases, BMMs were plated on plastic as a control. BMMs were treated with 10 ng/ml LPS, tumor necrosis factor–α (TNF–α), or a matching volume of vehicle to remain unstimulated (US). Cultures were maintained at 37°C with 5% CO_2_.

### ELISA and Griess Assay

ELISAs (eBioscience, San Diego, CA) measured TNF–α, IL–1β, and IL–6 concentrations in media and Griess reagent (Sigma–Aldrich) measured nitric oxide (NO) concentrations in media. The ELISAs and Griess assays were performed according to the manufacturer’s instructions. Concentrations were derived from standard curves. BMM density on gels determined whether cell density was influencing inflammatory mediator concentrations in media (S1 Fig). BMM density was calculated on all gels using ImageJ software.

### BMM Lysis

BMMs were lysed on ice for a minimum of 30 mins with 100 μl lysis buffer per well (25 mM Tris–HCl, 150 mM NaCl, 1% NP–40, 1 mM EDTA, 5% Glycerol, 1 mM phenylmethanesulfonylfluoride pH 7.4 containing a mixture of protease inhibitors (Complete Protease Inhibitor Cocktail Tablet, Roche, Indianapolis, IN)). Samples from the same condition were pooled for each biological replicate.

### NF–κB Translocation Assay

A Nucelar/Cytosol Fractionation Kit was purchased from BioVision (Milpitas, CA). Fractionation was performed according to the manufacturer’s instructions. Nuclear and cytosolic fractions were subjected to Western blotting and blots were probed for p–NF–κB p65.

### Western Blotting

Protein concentrations were measured with a bicinchoninic acid protein assay kit according to the manufacturer’s instructions (Sigma–Aldrich). Lysates were loaded (10 μg for GAPDH and 25–50 μg for all others) and separated on a 7.5% SDS–PAGE gel. Proteins were electrotransferred onto a PVDF membrane and blocked with blocking solution (5% non–fat dry milk in TBS containing 0.1% Tween 20) for 1.5 hrs at room temperature (RT). All primary antibodies were diluted 1:500 in blocking solution and blots were probed with indicated primary antibody overnight at 4°C. Blots were washed thrice and probed with HRP–conjugated secondary antibody for 1.5 hrs at RT. Blots were washed thrice and developed using ECL Western Blotting Substrate (Pierce, Rockford, IL). Chemiluminescent images were captured using a PXi 6 Touch gel documentation system (SynGene, Frederick, MD). GAPDH protein levels were used as controls for adequacy of equal protein concentrations. Band intensities for three or more biological replicates were measured with GeneTools software (SynGene) or Image J (NIH, Bethesda, MD). All bands were divided by GAPDH band intensity except for bands in the translocations assay. Band Intensities were further normalized to band intensities of 230 kPa gel, IκBα, or NF–κB as indicated in the figures. Graphs show the average of three or more biological replicates.

### Statistics

Data are a combination of three or more biological replicates. Graphs show mean ± SEM of combined data. Data was tested for normality using a D’Agostino and Pearson omnibus normality test. A parametric test was performed on data that passed the normality test, while a nonparametric test was performed on data that did not pass the normality test. Statistical tests are described in the figure legends. A singular horizontal grey line with brackets indicates that all conditions within the bracket are significantly different to each other. A single horizontal grey line without brackets indicates that only the conditions on the two endpoints are significantly different to each other. Multiple horizontal lines with brackets indicate multiple conditions are significantly different from each other. Conditions within the first horizontal line are significantly different to the conditions within the second horizontal line. *p<0.05, **p<0.01 and ***p<0.001 indicate significance for stimulated BMMs. while ^x^p<0.05, ^xx^p<0.01 and ^xxx^p<0.001 indicate significance for US BMMs. N_subtext_ indicates the number of cells or wells.

## Results

### Proinflammatory mediator secretion increases as substrate stiffness increases

**Fig 1:**
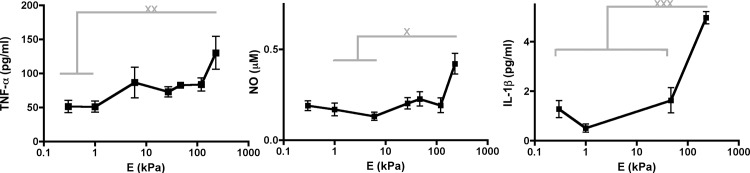
Proinflammatory mediator concentrations in media from US BMMs. Media from US BMMs grown on PA gels were subjected to ELISAs or Griess assays. Graphs show concentrations of TNF–α, NO, and IL–1β. Data from ELISAs were assessed using one–way ANOVA followed by Tukey’s multiple comparisons test and data from Griess assays were assessed using Kruskal–Wallis followed by Dunn’s multiple comparisons test. N_wells_≥7.

To evaluate the effect of substrate stiffness on BMMs, we evaluated proinflammatory mediator concentrations in media via ELISAs and Griess assays from steady–state or US BMMs grown on PDL–functionalized PA gels that mimicked a variety of tissue stiffnesses [10,21,22] (Fig 1). We found that TNF–α, IL–1β, and NO concentrations increased as substrate stiffness increased for US BMMs. However, US BMMs grown on any PA gel did not secrete IL–6. These data suggest that substrate stiffness regulates the secretion of specific inflammatory signatures in US BMMs.

**Fig 2:**
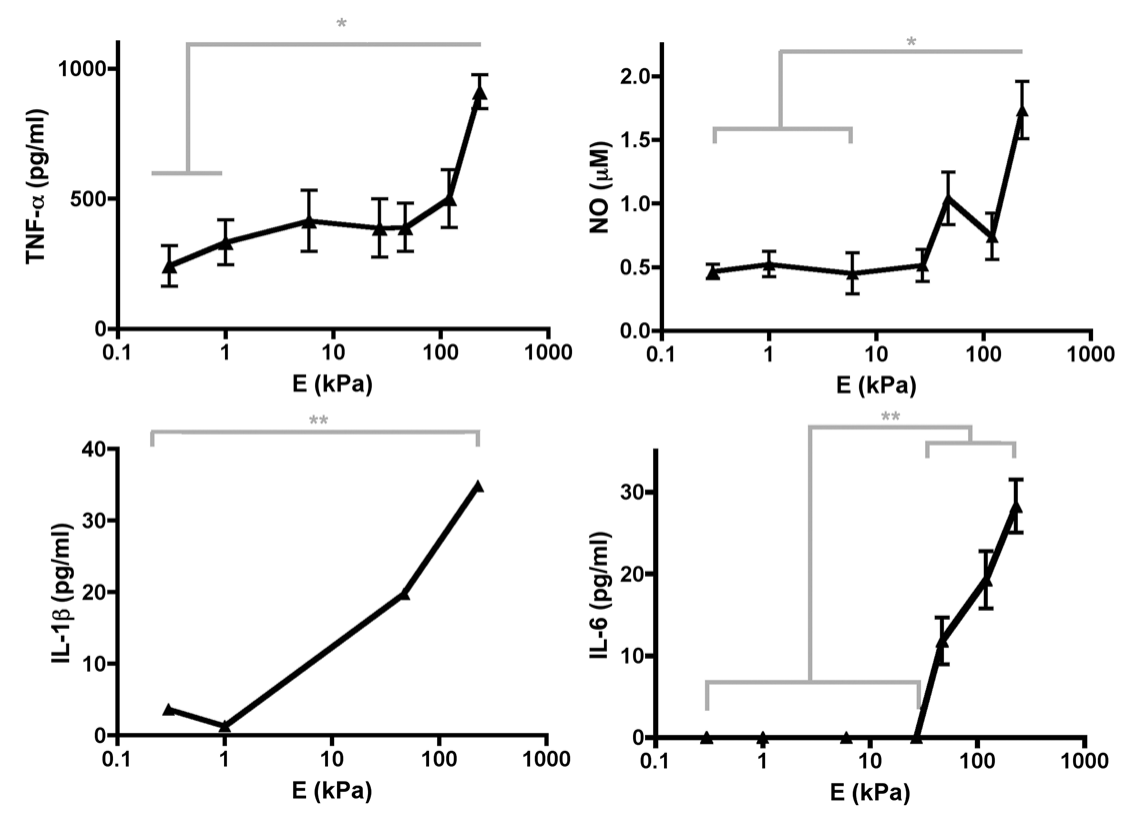
Proinflammatory mediator concentrations in media from LPS–stimulated BMMs. Media from LPS–stimulated BMMs grown on PA gels were subjected to ELISAs or Griess assays. Graphs show concentrations of TNF–α, IL–1β, NO, and IL–6. Data from ELISAs were assessed using one–way ANOVA followed by Tukey’s multiple comparisons test and data from Griess assays were assessed using Kruskal–Wallis followed by Dunn’s multiple comparisons test. N_wells_≥7.

**Fig 3:**
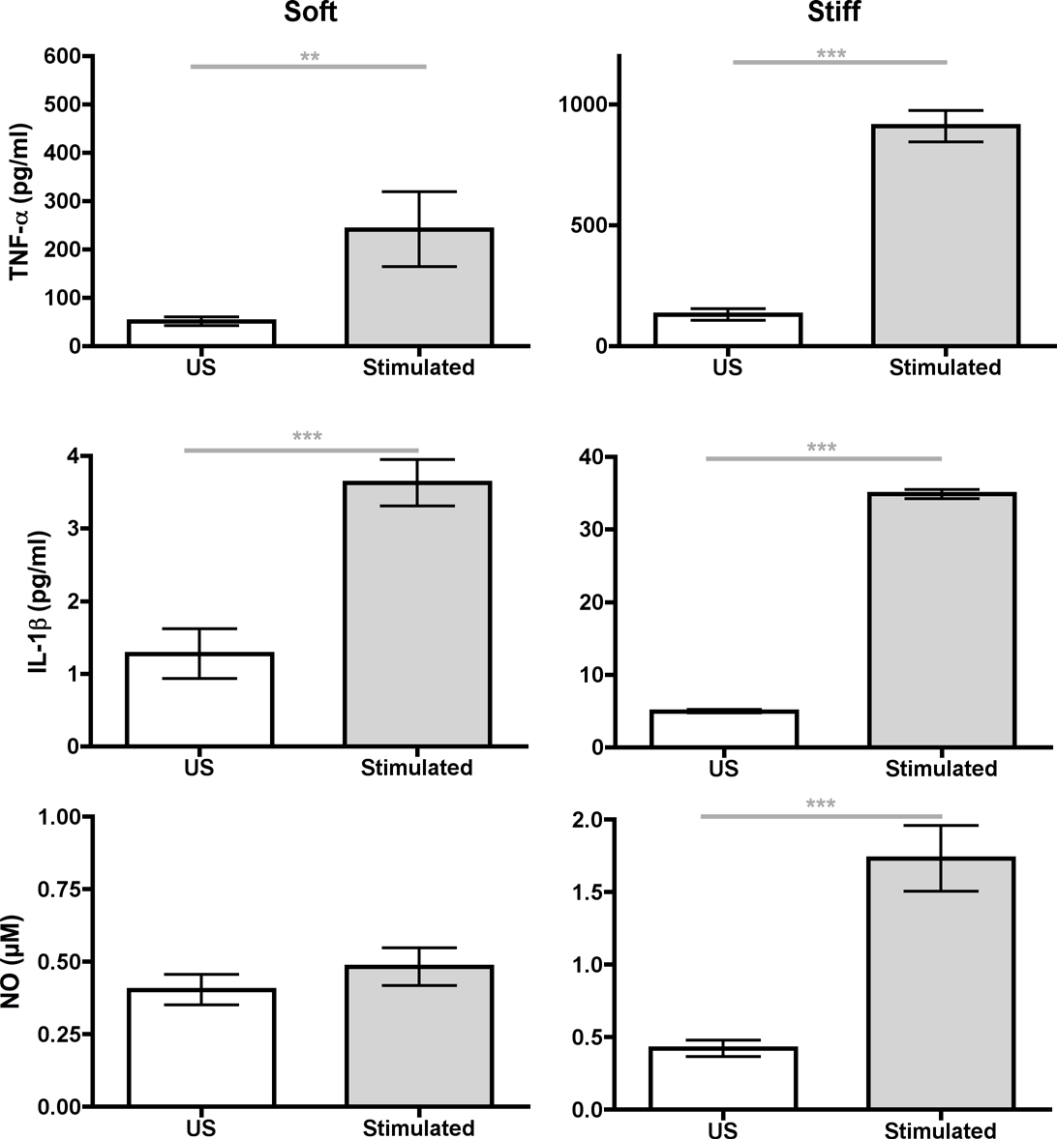
Comparison of proinflammatory mediator concentrations between US (*white bars*) and LPS–stimulated (*grey bars*). BMMs were grown on soft *(0*.*3 kPa*, *left graphs)* and stiff *(230 kPa*, *right graphs)* substrates. Data were assessed using Mann–Whitney U test. N_wells_≥7.

Next, we explored the effects of substrate stiffness on activated or stimulated macrophages (Fig 2). BMMs were grown on PDL–functionalized gels, glass, or tissue culture plastic and were stimulated with 10–20 ng/ml of LPS. As expected for the control, the presence of LPS increased NO and TNF–α secretion when BMMs were grown on glass or plastic (S2 Fig). Stimulated BMMs grown on PA gels had similar results to US BMMs grown on PA gels; i.e. TNF–α, IL–1β, IL–6, and NO concentrations increased as substrate stiffness increased (Fig 2). However, stimulated BMMs grown on PA gels had higher concentrations of TNF–α, IL–1β, and NO in media than US BMMs grown on PA gels (Fig 3). Plating density was the same for all conditions and BMM density was similar among all gels (S1 Fig). Therefore, BMM density did not account for differences in proinflammatory mediator concentrations in media.

**Fig 4:**
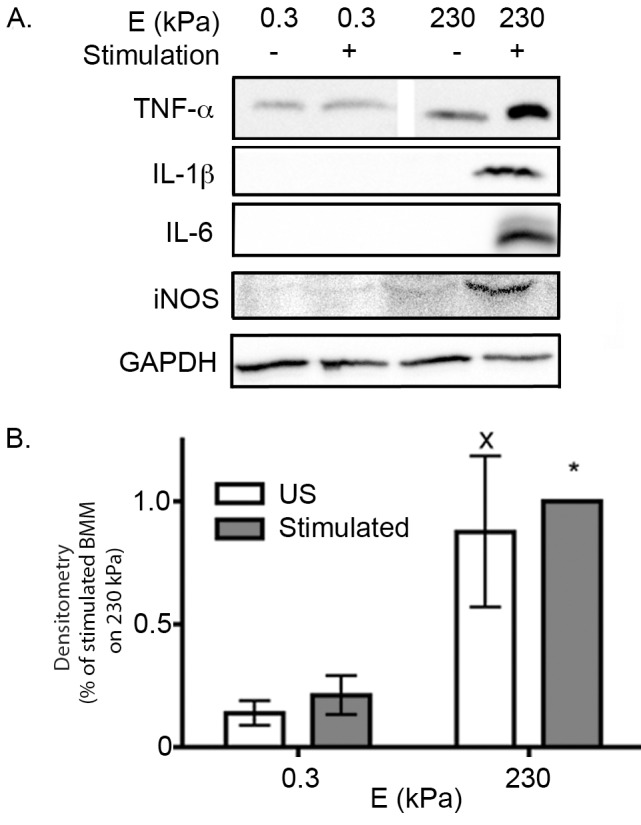
Proinflammatory mediator expression. Lysates from US and stimulated BMMs grown on PA gels were subjected to Western blotting. *(A)* Representative immunoblots. Blots were probed with (*from top to bottom*) rabbit anti–TNF–α, anti–IL–1β, anti–IL–6, anti–iNOS, or GAPDH antibodies. *(B)* Densitometry for anti–TNF–α. Data were assessed using one–way ANOVA followed by Tukey’s multiple comparisons test. Densitometry was not performed for blots probed for IL–1β, IL–6, iNOS because only stimulated BMMs on 230 kPa show protein expression. Three or more samples were subjected to Western blotting for each antibody.

To confirm our ELISA and Griess assay findings, intracellular proinflammatory mediator signatures were evaluated in BMMs grown on PDL–functionalized PA gels. Twenty–four hours after plating BMMs on gels, BMMs were lysed and lysates were subjected to Western blotting. Densitometry, which shows the average quantification for three or more replicates, demonstrates that stimulated BMMs expressed more TNF–α, IL–1β, IL–6, iNOS when grown on 230 kPa gels than on 0.3 kPa gels (Fig 4).

ELISAs, Griess assays, and Western blotting results suggest that stiff substrates promote proinflammatory mediator production. These data also confirm that stimulated BMMs had an increase in proinflammatory mediator production compared to US BMMs. In addition, these results suggest that proinflammatory mediator signatures in media were not a caveat of stiffness–regulated exocytosis.

### Proinflammatory mediator secretion increases as substrate stiffness increases when BMMs were stimulated with TNF–α

**Fig 5:**
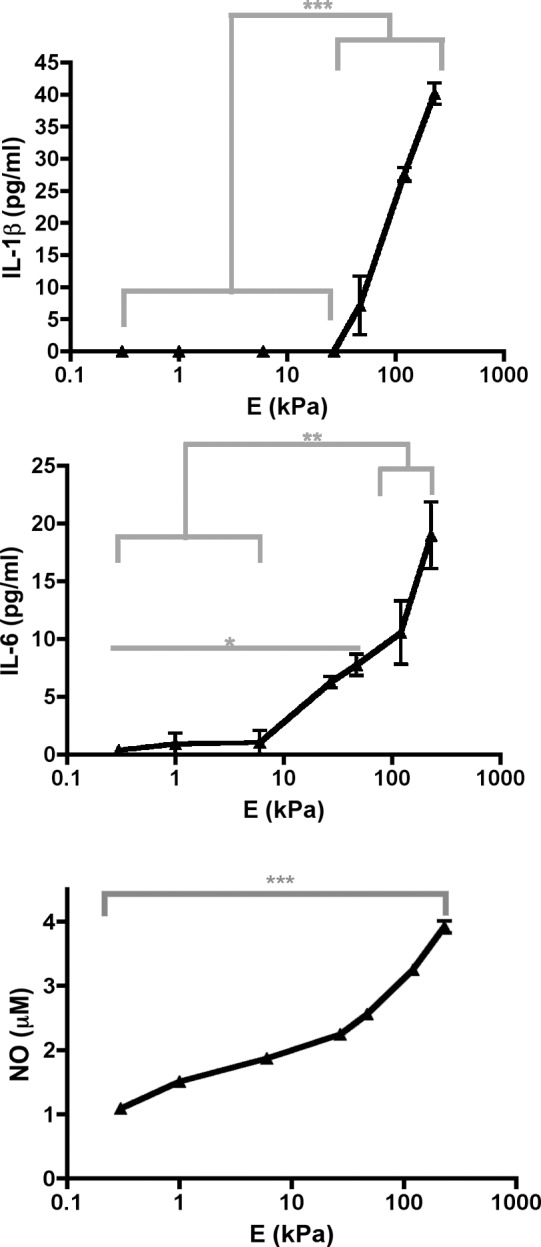
Proinflammatory mediator concentrations in media from TNF–α–stimulated BMMs. Media from TNF–α–stimulated BMMs grown on PA gels were subjected to ELISAs or Griess assays. Graphs show concentrations of (*from top to bottom*) IL–1β, IL–6, and NO. Data were assessed using one–way ANOVA followed by Tukey’s multiple comparisons test. N_wells_≥ 4.

**Fig 6:**
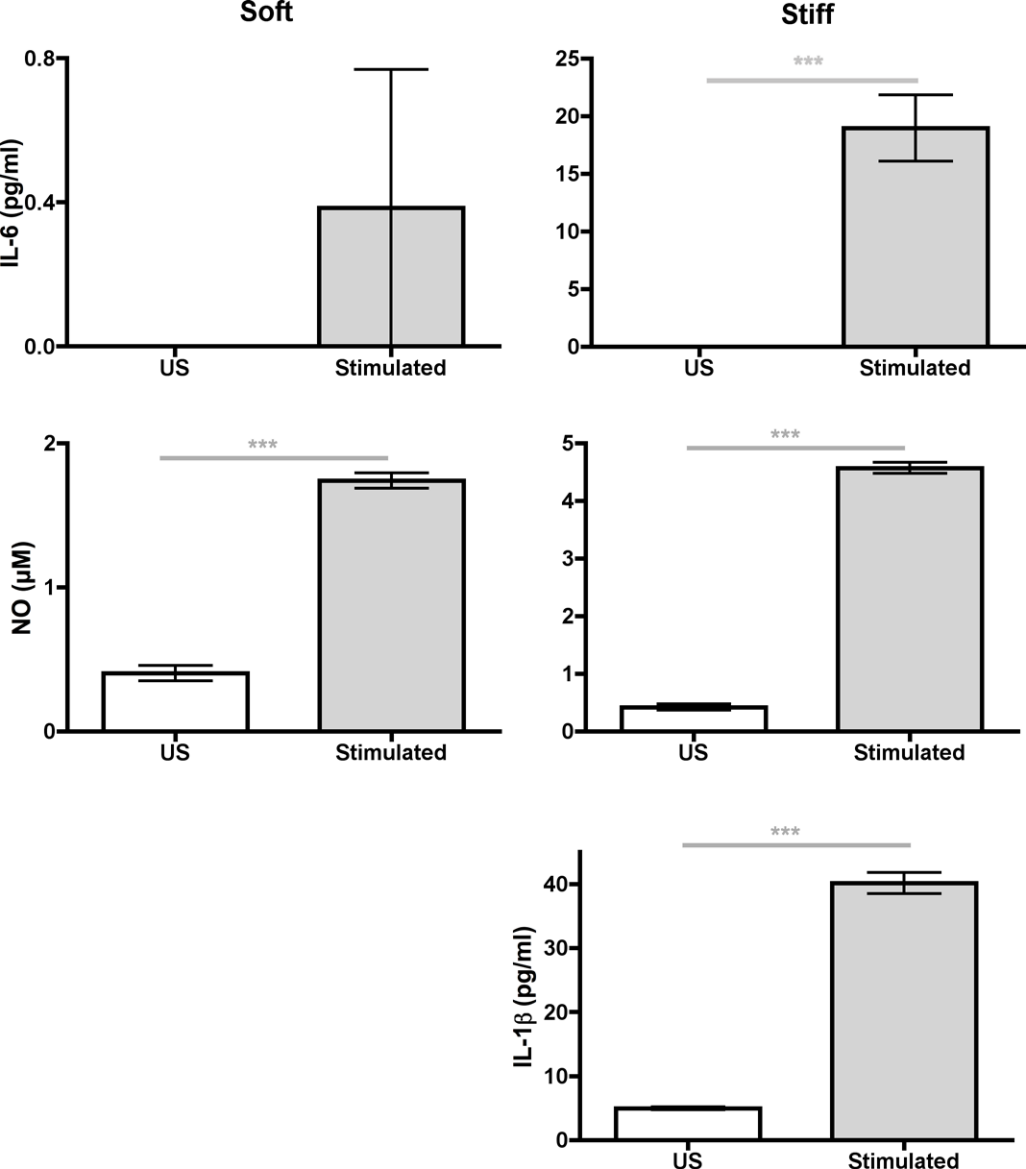
Comparison of proinflammatory mediator concentrations in media between US (*white bars*) and TNF–α–stimulated (*grey bars*) BMM. BMMs were grown on soft *(left graphs)* and stiff *(right graphs)* substrates. N_wells_≥7. Data were assessed using Mann–Whitney U test. N_wells_≥ 4.

We evaluated whether the type of biological stimulant had implications on stiffness–regulated proinflammatory activity. We stimulated BMMs grown on PDL–functionalized PA gels with 10 ng/ml of TNF–α. Like LPS–stimulated BMMs, TNF–α–stimulated BMMs had an increase in IL–1β, IL–6, and NO concentrations in media as substrate stiffness increased (Fig 5). In addition, TNF–α–stimulated BMMs had higher proinflammatory mediator concentrations in media on all gels compared to US BMMs (Fig 6). Therefore, TNF–α–stimulated BMMs responded similarly to substrate stiffness as LPS–stimulated BMMs (Figs 2, 3, 5 and 6).

### Proinflammatory activity is not directed by the extracellular matrix (ECM) ligand functionalized on gels

The ECM is a major contributor to the mechanical properties of tissues. ECM proteins such as collagen type I and laminin undergo major changes in distribution in diseased tissues [23,24] and stimulate macrophage activity [25]. In addition, ECM ligands can direct the cell’s response to substrate stiffness [26,27]. For instance, mesenchymal stems cells are directed towards osteogenesis when plated on substrates mimicking collagenous bone stiffness and functionalized with collagen type I or fibronectin [26], but this phenotype is eliminated when mesenchymal stem cells are plated on substrates mimicking collagenous bone stiffness and functionalized with collagen IV or laminin. Therefore, we hypothesized that the ECM ligand functionalized on gels directs stiffness–regulated proinflammatory mediator production.

**Fig 7:**
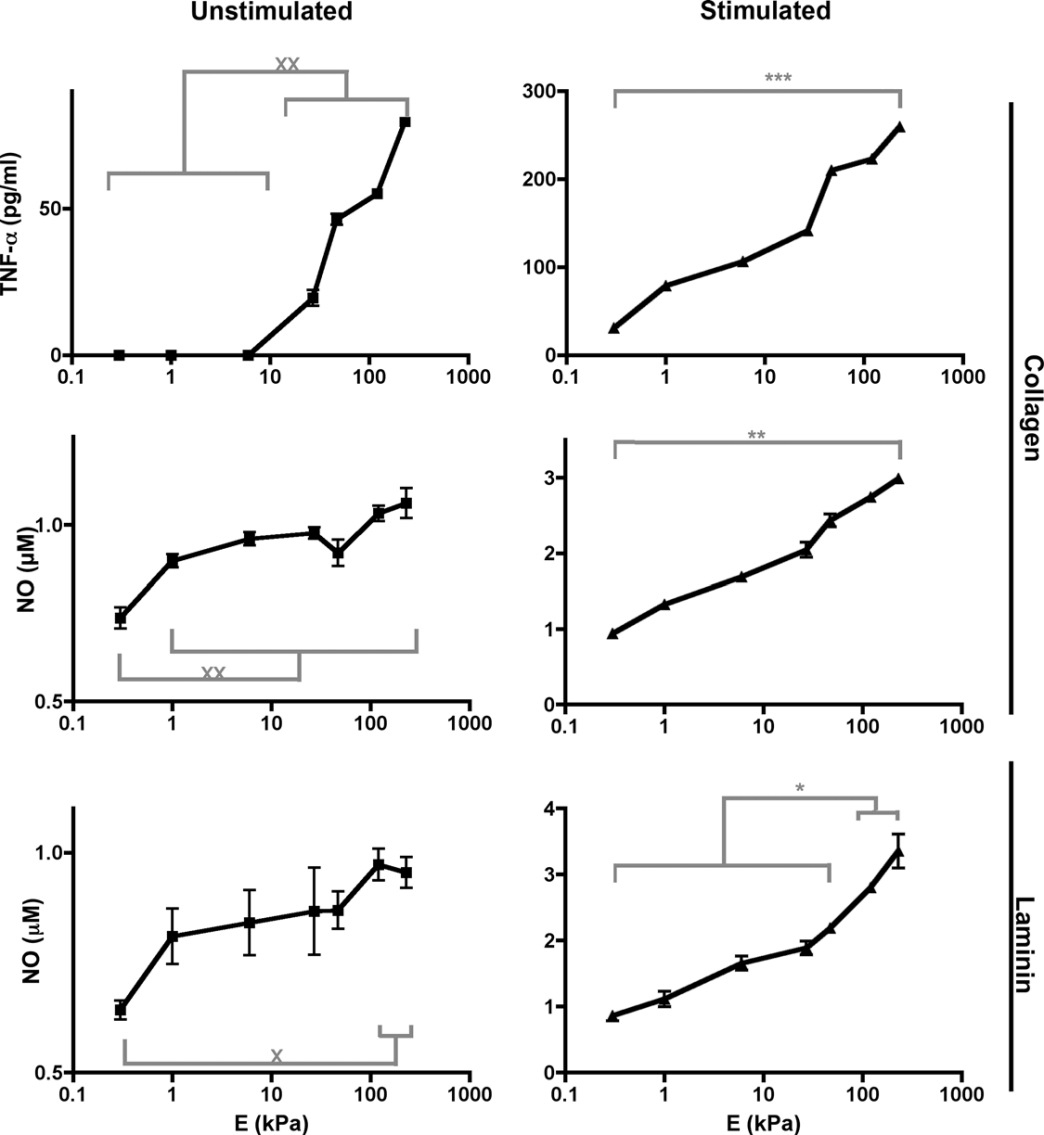
Proinflammatory mediator concentrations in media. US (*left graphs*) and stimulated (*right graphs*) BMMs were grown on collagen–or laminin–functionalized gels. Media were collected and subjected to ELISAs and Griess assays. Laminin experiments were performed in duplicate. Graphs show concentrations of TNF–α and NO. Data were assessed using one–way ANOVA followed by Tukey’s multiple comparisons test. N_wells_≥ 4.

**Fig 8:**
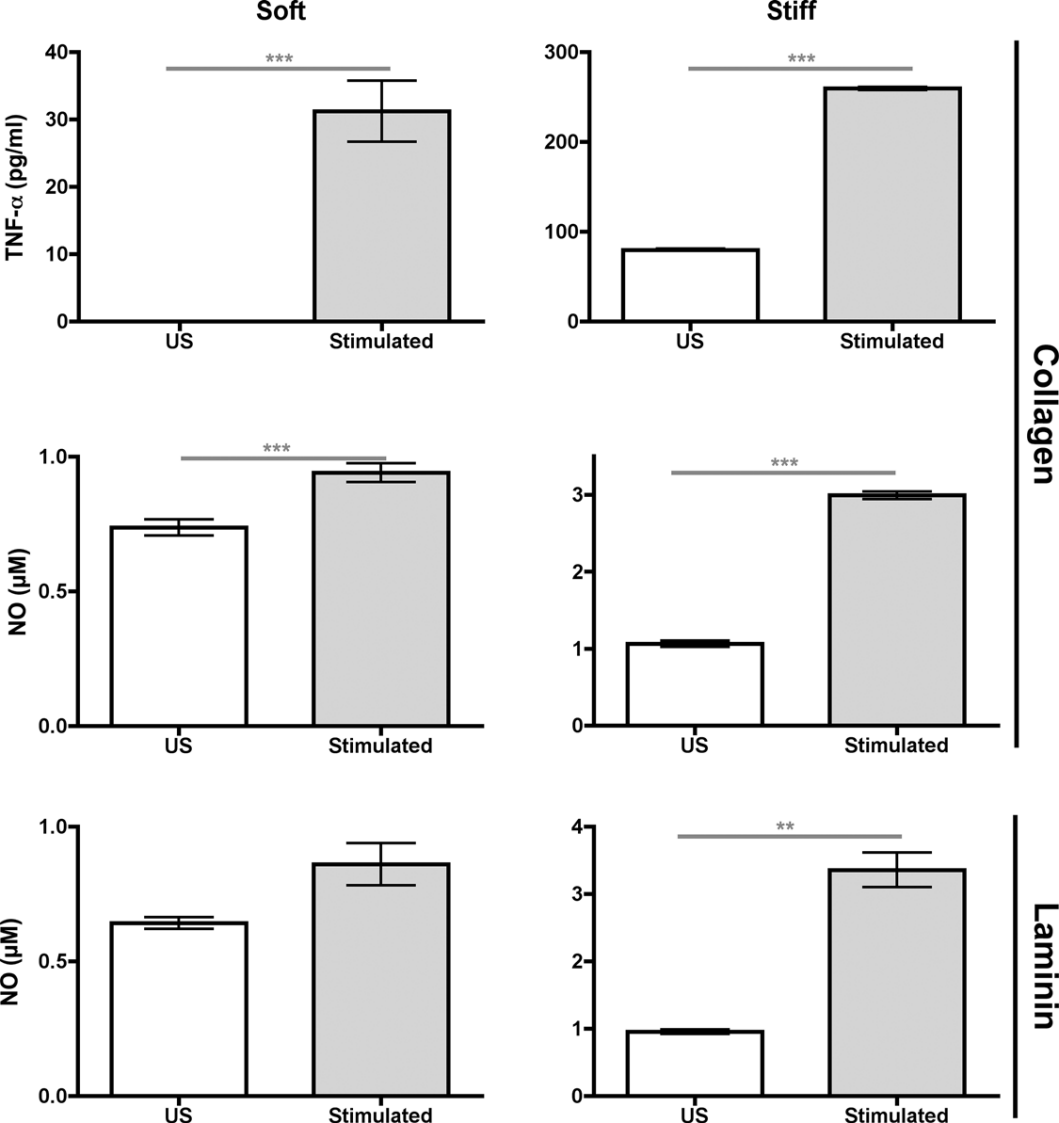
Comparison of proinflammatory mediator concentrations in media between US (*white bars*) and stimulated (*grey bars*) BMMs. BMMs were grown on collagen–or laminin–functionalized soft (*left graphs)* and stiff *(right graphs)* gels. Data were assessed using Mann–Whitney U test. N_wells_≥ 4.

To test our hypothesis, we evaluated the proinflammatory signatures of BMMs grown on gels functionalized with collagen type I (collagen) and laminin (Fig 7) instead of PDL (Figs 1–6). Similar to PDL–functionalized gels, we found that US and stimulated BMMs had increased concentrations of TNF–α and NO in media as substrate stiffness increased independent of the ligand that is functionalized on gels. In addition, stimulated BMMs had higher proinflammatory mediator concentrations in media when compared to US BMMs on all gels (Fig 8). Therefore, stiffness–regulated proinflammatory mediator production occurs when gels are functionalized with or without ECM.

### TLR4 signal transduction increases as substrate stiffness increases

Our data indicate that LPS enhanced proinflammatory mediator production of BMMs grown on stiff substrates (Figs 2 and 3). We therefore speculated that the activity of the LPS receptor TLR4 is affected by substrate stiffness. To determine the effect of substrate stiffness on the TLR4 signaling pathway, we first evaluated TLR4 expression in US and LPS–stimulated BMMs grown on collagen–PDL–functionalized 0.3 and 230 kPa gels. These two extreme stiffnesses were chosen because BMMs grown on these PA gels showed the most signficant differences in proinflammatory mediator concentrations and cell morphology remained homogenous on both 0.3 and 230 kPa gels [28].

**Fig 9:**
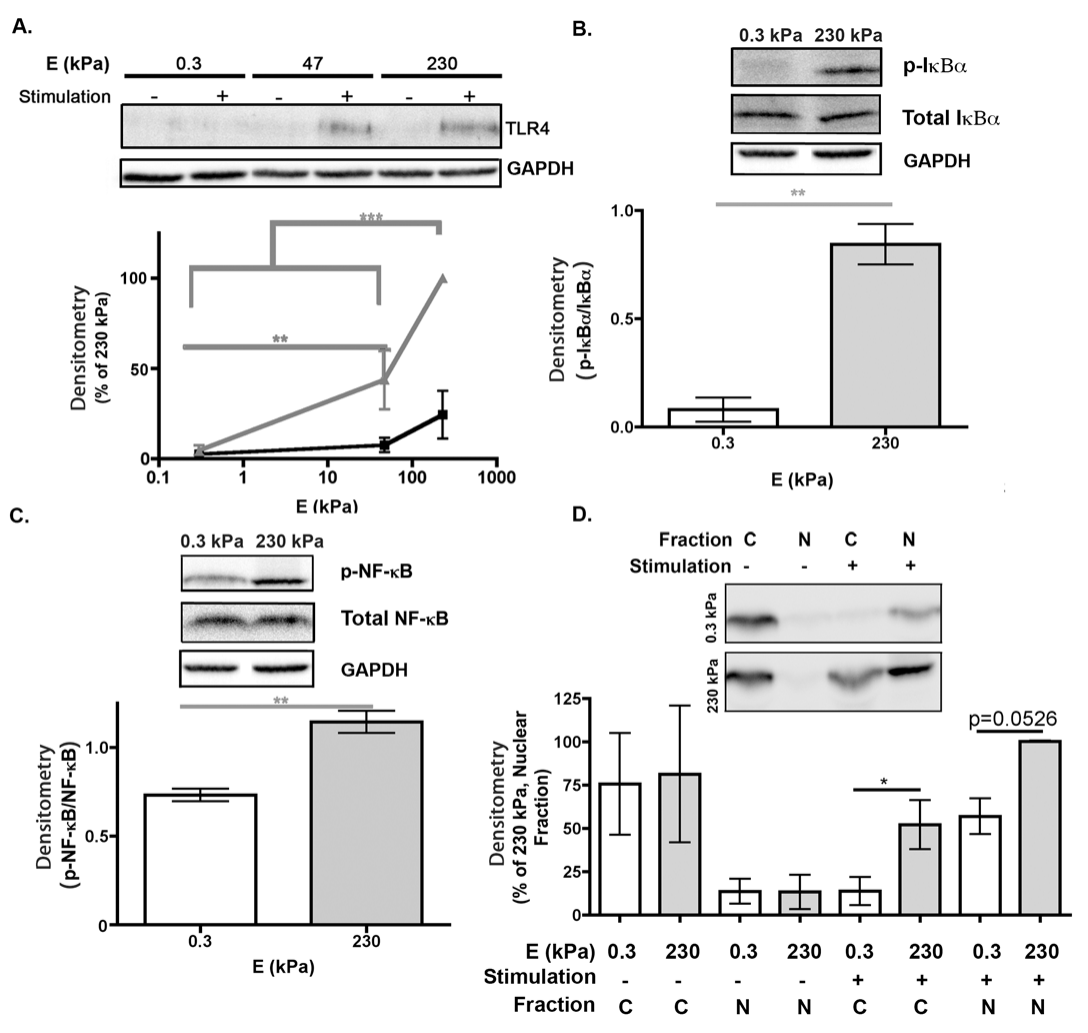
TLR4 signal transduction on PA gels. *(A)* Representative TLR4 immunoblot (*top*) and densitometry (*bottom*). Lysates from US and stimulated BMMs grown on 0.3, 47, or 230 kPa gels were subjected to Western blotting. Blots were probed with (*from top to bottom*) rabbit anti–TLR4 or anti–GAPDH antibodies. For densitometry, data are a percentage of 230 kPa band intensities. Data were assessed using one–way ANOVA followed by Tukey’s multiple comparisons test. *(B)* Representative IκBα immunoblot (*top*) and densitometry (*bottom*). Blots were probed with (*from top to bottom*) anti–p–IκBα, anti–IκBα, or anti–GAPDH antibodies. For densitometry, data are a ratio of p–IκBα/IκBα. Data were assessed using unpaired t test with Welch’s correction. *(C)* Representative NF–κB (*top*) immunoblot and densitometry (*bottom*). Blots were probed with (*from top to bottom*) anti–p–NF–κB, anti–NF–κB and anti–GAPDH antibodies. For densitometry, data are a ratio of p–NF–κB/NF–κB. Data were assessed using unpaired t test with Welch’s correction. *(D)* Representative p–NF–κB immunoblot for cytosolic (C) and nuclear (N) fractions (*top*) and densitometry (*bottom*). Blots were probed with anti–p–NF–κB antibody. For densitometry, data are a percentage of 230 kPa band intensities. Data were assessed using unpaired t test with Welch’s correction. Three or more samples were subjected to Western blotting for each antibody.

US and stimulated BMM lysates were subjected to Western blotting and blots were probed for TLR4. Blots and densitometry, which show the average quantification of three or more blots, demonstrate that TLR4 expression was higher for stimulated BMMs grown on 230 kPa than on 0.3 kPa gels (Fig 9A). This suggests that TLR4 expression is regulated by substrate stiffness.

Next, we evaluated the phosphorylation of downstream signaling proteins IκBα and NF–κB p65 as well as translocation of p–NF–κB p65 in LPS–stimulated BMMs grown on 0.3 amd 230 kPa gels [29]. Following TLR4 activation with LPS, TLR4 recruits the cytoplasmic adaptor molecule, MyD88 in the MyD88–dependent TLR4 signaling pathway. MyD88 interacts with IRAK, which leads to activations of another adaptor molecule TRAF6. In turn, MAPK kinases and the IKK complexes are activated. The IKK complex phosphorylates IκBα and NF–κB p65 [30]. p–IκBα is ubiquitinated and then is targeted to the proteasome. p–IκBα degradation liberates p–NF–κB p65 from the IκB complex to allow p–NF-κB p65 nuclear translocation [29,31,32].

First, we investigated IκBα phosphorylation (Ser32/36) via Western blotting. Blots were probed for p-IκBα and total IκBα (Fig 9B). Blots and densitometry, which show the average quantification of three or more blots, demonstrate that there was a higher amount of p–IκBα for stimulated BMMs grown on 230 kPa gels than on 0.3 kPa gels. Second, we investigated NF–κB p65 phosphorylation (Ser536). Blots were probed for p–NF–κB and total NF–κB p65 (Fig 9C). Blots and densitometry, which show the average quantification of three or more blots, demonstrate that there was a higher amount of p–NF–κB for stimulated BMMs grown on 230 kPa gels than on 0.3 kPa gels (Fig 9C). Third, nuclear translocation was evaluated by measuring the amount of p–NF–κB in the nucleus and cytosol. Nucelar and cytosol were fractionated and subjected to Western blotting. Blots were probed for p–NF–κB. Blots and densitometry, which show the average quantification of three or more blots, demonstrate that US BMMs grown on 0.3 and 230 kPa gels had equal amounts of p–NF–κB in the cytosol and nucleus (Fig 9D). However, stimulated BMMs had higher amounts of p–NF–κB in the cytosol and nucleus when grown on 230 kPa gels than 0.3 kPa gels. These results indicate that stiffer substrates promote p–NF–κB translocation from the cytosol to the nucleus when LPS is present. TLR4, NF–κB, and IκBα evidence suggests that substrate stiffness regulation of proinflammation is mediated through TLR4 activity.

**Fig 10:**
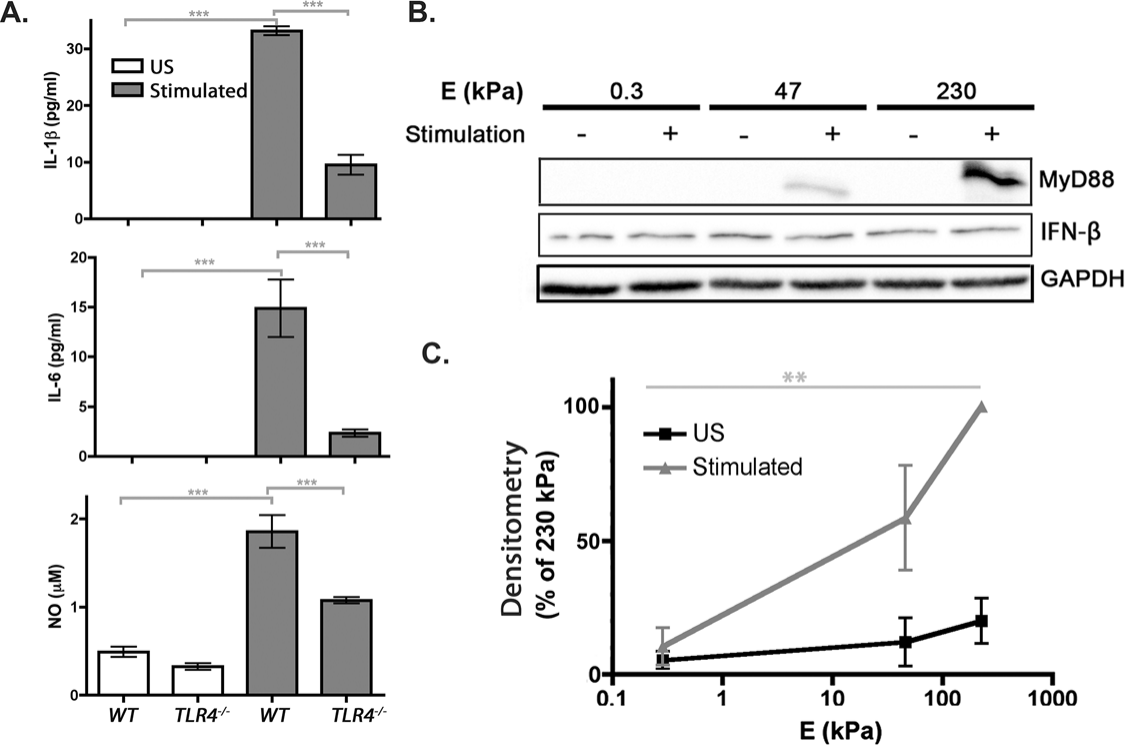
MyD88-dependent TLR4 signal transduction. *(A)* TLR4–deficient BMMs grown on gels. Media from US (*white bars*) and stimulated (*grey bars*) TLR4–deficient (*TLR4* ^–/–^) and WT BMMs grown on 230 kPa gels were subjected to ELISAs and Griess assays. Graphs show concentrations of (*from top to bottom*) IL–1β, IL–6, and NO. Data from ELISAs were assessed using Kruskal–Wallis followed by Dunn’s multiple comparisons test and data from Griess assays were assessed using one–way ANOVA followed by Tukey’s multiple comparisons test. N_wells_≥ 21. *(B)* Representative MyD88 (*top*) and IFN–β (*middle*) immunoblots. Lysates from US and stimulated BMM grown on 0.3, 47, and 230 kPa gels were subjected to Western blotting. Blots were probed with (*from top to bottom*) anti–MyD88, anti–IFN–β, and anti–GAPDH antibodies. *(C)* Densitometry. Data are a percentage of 230 kPa band intensities. Data were assessed using one–way ANOVA followed by Tukey’s multiple comparisons test. Densitometry for IFN–β was not performed because no visual changes were observed. Three or more samples were subjected to Western blotting.

We confirmed the effect of substrate stiffness on TLR4 activity by harvesting BMMs from B6.B10ScN–*Tlr4* ^*lps–del*^/JthJ mice, which have a spontaneous mutation that removes the *Tlr4* coding sequence, and growing these TLR4–deficient BMMs on PDL–functionalized 230 kPa gels. The proinflammatory mediator concentrations in the media were measured and compared to WT BMMs grown on 230 kPa gels. Neither IL–6 nor IL–1β were detected in US WT or TLR4–deficient BMMs media, but stimulated TLR4–deficient BMMs had a significant decrease in IL–1β, IL–6, and NO secretion compared to stimulated WT BMMs (Fig 10A). Together, these data suggest that proinflammatory mediator production by LPS–stimulated BMMs on stiff substrates is regulated by TLR4 activity. Interestingly, secretion of proinflammatory mediators was not completely abolished when TLR4 was depleted, suggesting that another LPS receptor may be contributing to the observed proinflammatory mediator production on stiff substrates.

### MyD88 expression increases as substrate stiffness increases

Because TLR4 activation can initiate one of two non–redundant pathways, the MyD88–dependent and MyD88–independent pathways, both pathways were evaluated (Fig 10B and 10C). Here, MyD88 and IFN–β expression was evaluated to further investigate MyD88–dependent and MyD88–independent pathway, respectively. Contrasting to the MyD88–dependent signaling, MyD88–independent signaling involves TRIF and TRAM, which induce IRF3 activation and IFN–β production [33,34]. (Fig 9) suggests that the MyD88–dependent pathway is involved in stiffness–regulated proinflammation since MyD88 recruitment is upstream of IκBα phosphorylation events. Again, US and stimulated BMMs were grown on PDL–or collagen–functionalized 0.3 and 230 kPa gels and lysed after 24 hrs. Lysates were subjected to Western blotting and blots were probed with the indicated antibodies. In agreement with Fig 9, blots and densitometry, which show the average quantification of three or more blots, demonstrate that MyD88 expression was higher for stimulated BMMs grown on 230 kPa than on 0.3 kPa gels (Fig 10B and 10C). In addition, blots show that IFN–β expression did not change with changes in substrate stiffness (Fig 10B). Collectively, these data suggest that substrate stiffness regulates MyD88–dependent but not MyD88–independent TLR4 signal transduction.

## Discussion

### Substrate stiffness regulates proinflammatory mediator production

Our results indicate that substrate stiffness regulates proinflammatory mediator production. First, our results show that US and stimulated macrophages had an increase in proinflammatory mediator production when substrate stiffness increased, with significant increases on gels that were ≥47 kPa (Figs 1–8, respectively). Tissues that are within this range of stiffness include human spinal cord (89 kPa), human collagenous bone (<34 kPa), atherosclerotic tissue (54–290 kPa), human thyroid cancer tissue (45 kPa), rodent myocardial infarct tissue (55 kPa), and rodent skeletal muscle (100 kPa) [3,10,35,36,37]. Therefore, our stiffness range that shows significant proinflamamtory mediator production encompasses both healthy and diseased tissues. Second, our results show that the presence of LPS or TNF–α in BMM cultures had a larger degree of proinflammatory mediator production than US BMMs when grown on soft or stiff substrates (Figs 3, 6 and 8). This suggests that mechanical properties of soft tissues in the range of 0.3–230 kPa can enhance the biological stimulation of macrophages. Third, we found that the presence of LPS or TNF–α in BMM cultures grown on stiff substrates had a higher concentration of NO and TNF–α than BMMs grown on plastic or glass (S2 Fig, and Figs 1–6). In fact, NO and TNF–α concentrations for BMMs grown on plastic or glass was about 0.75 μM and 500 pg/ml, respectively (S2 Fig), and NO and TNF–α concentrations for BMMs grown on 230 kPa gels was about 1.75 and 800 pg/ml, respectively (Figs 2, 3, 6 and 7). With plastic and glass stiffness being out of the range of biological soft tissues, these data indicate that biological stimuli and elastic substrates in the range of soft biological tissues can interact synergistically to enhance proinflammatory phenotypes of macrophages.

Like the present study, previous reports have found that macrophages are responsive to elastic substrates. However, our results mostly disagree with previous reports. Unlike our report, Blakney *et al*. found that macrophages were overall unresponsive to substrate stiffness when stimulants were absent [13]. Discrepancy between our study and their study could be based on the range of substrate stiffness (130–840 kPa versus 0.3–230 kPa) and the type of substrate (PEG–RGD versus PA) utilized in each study. Nevertheless, like our study, Blakney *et al*. found that stimulated macrophages had an increase in cytokine production with an increase in substrate stiffness. In another study, Patel *et al*. showed that TNF–α production for RAW264.7 and U937 cells was inversely proportional to substrate stiffness, which ranged from 0.3–76.8 kPa [11]. Also, Irwin *et al*. found that human macrophages differentiated from THP–1 cells also had a decrease in TNF–α concentrations as substrate stiffness increased from 1.4 to 348 kPa [12]. The differences between our results and their results could be based on differences in the cell type (primary BMMs versus RAW264.7, THP-1, and U937). For example, Patel *et al*. found that TNF–α concentrations ranged from 30–70 pg/ml and 1250–2750 pg/ml for RAW264.7 and U937, respectively. In our study, TNF–α concentrations for LPS–stimulated primary macrophages ranged from 250–1000 pg/ml. These differences in the range of TNF–α concentrations indicate that cell types experience particular responses to LPS when grown on elastic substrates.

### Substrate stiffness regulates TLR4

Other studies have suggested that substrate stiffness regulates TLR4 activity. For example, Ishihara *et al*. found that stiff substrates enhanced NF–κB translocation and transcriptional activity, which initiates the production of proinflammatory mediators [38]. Here, we start to build further upon this mechanism by showing mechanical properties of substrates regulate specific avenues of TLR4 activity.

We show that various downstream signaling molecules of the TLR4 signaling cascade respond to substrate stiffness. Specifically, we found that stiff substrates enhanced TLR4 and MyD88 expression, IκBα phosphorylation, and NF–κB p65 phosphorylation and translocation (Figs 9 and 10). We speculate that an increase in TLR4 expression resulted in an increase in the downstream signaling events of TLR4, i.e. MyD88 expression, IκBα phosphorylation, and NF–κB p65 phosphorylation and translocation. In future experiments, we want to identify whether other downstream MyD88–dependent signaling events are affected by substrate stiffness in order to generate a complete stiffness–regulated TLR4 signaling cascade. Specifically, we want to know the effect of substrate stiffness on the IKK complex phosphorylation, p–IκBα ubiquitination, and targeting of NF–κB p65 to determine whether the classical NF–κB activation cascade is triggered by stiff substrates. In addition, it is possible that structure and function within the nucleus is affected by substrate stiffness resulting in an increase in on TLR4 expression [39]. Therefore, nuclear structure and posttranscriptional and transcriptional events of TLR4 activity also need further investigation.

## Conclusion

Previous reports described the effects of substrate stiffness on stem cell [35,37], fibroblast [40], cardiac muscle [35,41], and neuronal cell [42,43] behavior, but limited studies have investigated the effects of tissue stiffness on macrophage function. Here, we show that stiff substrates promote proinflammatory mediator production and TLR4 activity in BMMs independent of ECM functionalization and the stimulant present.

## Supporting Information

S1 Fig BMM density. BMM density for stimulated (*triangular points*) and US (*square points*) BMMs grown on 0.3–230 kPa gels was measured. Three images per well were analyzed and averaged for PDL–functionalized gels. Dividing the cell–number average for each well by the cell–number average of US cells grown on 0.3 kPa normalized the data and accounted for plating variability between each individual experiment. N_images_≥7.
(TIF)

S2 Fig BMM activity on plastic or glass. BMMs were grown on tissue culture plastic or glass cover slips. Proinflammatory mediator secretion was measured via ELISA.
(TIF)
