# Supplementary figures and images for: Substrate Stiffness Regulates Proinflammatory Mediator Production through TLR4 Activity in Macrophages
Source: PLoS One. 2015 Dec 28;10(12):e0145813. doi: 10.1371/journal.pone.0145813 (PMC4692401; doi:10.1371/journal.pone.0145813)

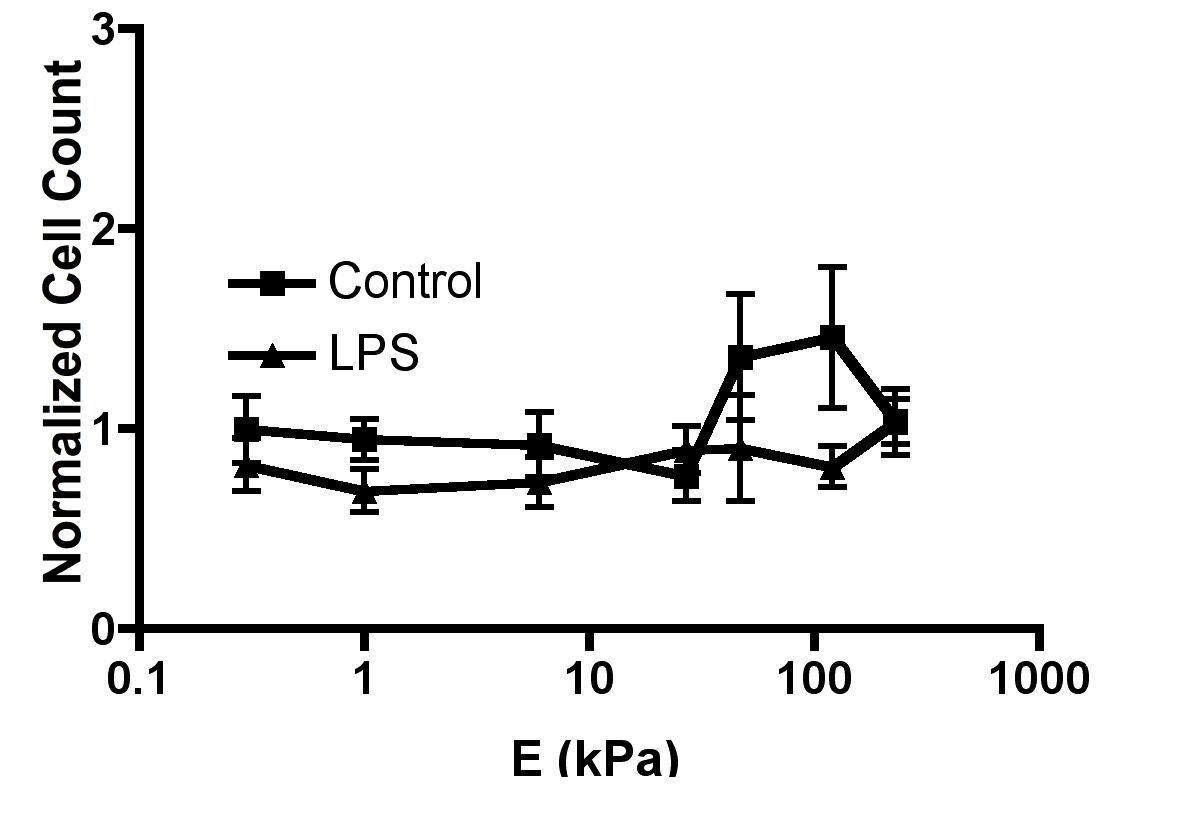

Supplement: S1 Fig — BMM density for stimulated (triangular points) and US (square points) BMMs grown on 0.3–230 kPa gels was measured. Three images per well were analyzed and averaged for PDL–functionalized gels. Dividing the cell–number average for each well by the cell–number average of US cells grown on 0.3 kPa normalized the data and accounted for plating variability between each individual experiment. Nimages≥7. (TIF) [file pone.0145813.s001.tif]

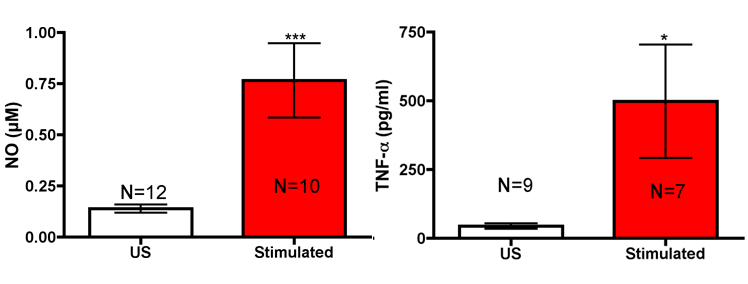

Supplement: S2 Fig — BMMs were grown on tissue culture plastic or glass cover slips. Proinflammatory mediator secretion was measured via ELISA. (TIF) [file pone.0145813.s002.tif]
